# A comparison of three thromboprophylaxis regimens in critically ill COVID-19 patients: An analysis of real-world data

**DOI:** 10.3389/fcvm.2022.978420

**Published:** 2022-08-16

**Authors:** Ahmed Alrashed, Peter Cahusac, Yahya A. Mohzari, Reem F. Bamogaddam, Mashael Alfaifi, Maya Mathew, Bashayer F. Alrumayyan, Basmah F. Alqahtani, Amjad Alshammari, Kholud AlNekhilan, Aljawharah Binrokan, Khalil Alamri, Abdullah Alshahrani, Safar Alshahrani, Ahmad S. Alanazi, Batool M. Alhassan, Ali Alsaeed, Wedad Almutairi, Asma Albujaidy, Lama AlJuaid, Ziyad S. Almalki, Nehad Ahmed, Hamdan N. Alajami, Hala M. Aljishi, Mohammed Alsheef, Saleh A. Alajlan, Faisal Almutairi, Atheer Alsirhani, Manayer Alotaibi, Melaf A. Aljaber, Hammam A. Bahammam, Hussain Aldandan, Abdulaziz S. Almulhim, Ivo Abraham, Ahmad Alamer

**Affiliations:** ^1^Administration of Pharmaceutical Services, Main Hospital, King Fahad Medical City, Riyadh, Saudi Arabia; ^2^Pharmacology and Biostatistics/Comparative Medicine, Alfaisal University College of Medicine, King Faisal Specialist Hospital and Research Centre, Riyadh, Saudi Arabia; ^3^Clinical Pharmacy Department, King Saud Medical City, Riyadh, Saudi Arabia; ^4^Department of Clinical Pharmacy, Almoosa Specialist Hospital, Al-Ahasa, Saudi Arabia; ^5^Department of Neurology, King Fahad Medical City, Riyadh, Saudi Arabia; ^6^Pharmacy College, Shaqra University, Riyadh, Saudi Arabia; ^7^Department of Clinical Pharmacy Service, Prince Mohammed Bin Abdulaziz Hospital, Riyadh, Saudi Arabia; ^8^Pharmacy College, Princess Nourah Bint Abdulrahman University, Riyadh, Saudi Arabia; ^9^Department of Clinical Pharmacy, College of Pharmacy, Prince Sattam Bin Abdulaziz University, Al-Kharj, Saudi Arabia; ^10^Research Center, King Fahad Medical City, Riyadh, Saudi Arabia; ^11^Medicine Department, King Fahad Medical City, Riyadh, Saudi Arabia; ^12^Department of Pediatric Dentistry, King Fahad Medical City, Riyadh, Saudi Arabia; ^13^College of Medicine, King Saud Bin Abdulaziz University for Health Sciences, Riyadh, Saudi Arabia; ^14^Department of Pharmacy Service, Prince Mutib Bin Abdulaziz Hospital, Sakaka, Saudi Arabia; ^15^Department of Pediatric Dentistry, College of Dentistry, King Abdulaziz University, Jeddah, Saudi Arabia; ^16^Department of Pharmacy, Alahsa Hospital, Al-Ahsa, Saudi Arabia; ^17^Department of Pharmacy Practice, College of Clinical Pharmacy, King Faisal University, Al-Ahsa, Saudi Arabia; ^18^Department of Pharmacy Practice and Science, College of Pharmacy, University of Arizona, Tuscon, AZ, United States

**Keywords:** thromboprophylaxis doses, critically ill patients, COVID-19, mortality, thromboprophylaxis

## Abstract

**Introduction:**

Thrombotic complications of coronavirus disease 2019 (COVID-19) have received considerable attention. Although numerous conflicting findings have compared escalated thromboprophylaxis doses with a standard dose to prevent thrombosis, there is a paucity of literature comparing clinical outcomes in three different anticoagulation dosing regimens. Thus, we investigated the effectiveness and safety profiles of standard, intermediate, and high-anti-coagulation dosing strategies in COVID-19 critically ill patients.

**Methodology:**

This retrospective multicenter cohort study of intensive care unit (ICU) patients from the period of April 2020 to August 2021 in four Saudi Arabian centers. Inclusion criteria were age ≥ 18 years, diagnosis with severe or critical COVID-19 infection, and receiving prophylactic anticoagulant dose within 24–48 h of ICU admission. The primary endpoint was a composite of thrombotic events, with mortality rate and minor or major bleeding serving as secondary endpoints. We applied survival analyses with a matching weights procedure to control for confounding variables in the three arms.

**Results:**

A total of 811 patient records were reviewed, with 551 (standard-dose = 192, intermediate-dose = 180, and high-dose = 179) included in the analysis. After using weights matching, we found that the standard-dose group was not associated with an increase in the composite thrombotic events endpoint when compared to the intermediate-dose group {19.8 vs. 25%; adjusted hazard ratio (aHR) =1.46, [95% confidence of interval (CI), 0.94–2.26]} or when compared to high-dose group [19.8 vs. 24%; aHR = 1.22 (95% CI, 0.88–1.72)]. Also, there were no statistically significant differences in overall in-hospital mortality between the standard-dose and the intermediate-dose group [51 vs. 53.4%; aHR = 1.4 (95% CI, 0.88–2.33)] or standard-dose and high-dose group [51 vs. 61.1%; aHR = 1.3 (95% CI, 0.83–2.20)]. Moreover, the risk of major bleeding was comparable in all three groups [standard vs. intermediate: 4.8 vs. 2.8%; aHR = 0.8 (95% CI, 0.23–2.74); standard vs. high: 4.8 vs. 9%; aHR = 2.1 (95% CI, 0.79–5.80)]. However, intermediate-dose and high-dose were both associated with an increase in minor bleeding incidence with aHR = 2.9 (95% CI, 1.26–6.80) and aHR = 3.9 (95% CI, 1.73–8.76), respectively.

**Conclusion:**

Among COVID-19 patients admitted to the ICU, the three dosing regimens did not significantly affect the composite of thrombotic events and mortality. Compared with the standard-dose regimen, intermediate and high-dosing thromboprophylaxis were associated with a higher risk of minor but not major bleeding. Thus, these data recommend a standard dose as the preferred regimen.

## Introduction

In addition to pulmonary manifestations of coronavirus 2019 (COVID-19) (1, 2), there is increasing concern about COVID-19-related extra-pulmonary complications, including thrombotic complications (3). Following emerging data, investigators paid more attention to the hypercoagulability state seen in COVID-19 cases that can result in the development of microthrombi in pulmonary microvasculature, deep vein thrombosis (VTE), and pulmonary embolisms (PE) (4). Initial studies from the early days of COVID-19 found a trend of thrombotic events in COVID-19 patients which prompted a flood of research in this area (5, 6). The incidence of thrombotic events was broadly inconsistent, with reports claiming that up to 69% of COVID-19 patients in a specific population were affected, despite the use of thromboprophylaxis (5–8). Compared to non-COVID-19 patients, previous studies showed COVID-19 patients have a greater risk of venous thromboembolism (VTE) 11.7 vs. 4.8% (9). According to meta-analyses, the incidence of VTE in intensive care unit (ICU)-admitted patients is higher than in those admitted to the general ward, with rates of 31 and 7%, respectively (10, 11). Such coagulation dysfunction has been associated with poor prognosis and negative outcomes, with 40% dying in those who developed VTE in the ICU (12, 13). Given high VTE occurrence reports and its unfavorable prognosis post-COVID-19 infection (14), some experts, agencies, and scientific committees advocated for increased thromboprophylaxis doses to be considered despite the absence of randomized evidence (15–18).

A comprehensive understanding of COVID-19 pathogenesis is still unclear. However, with available data related to the pathology of venous thromboembolism, severe acute respiratory syndrome coronavirus-2 (SARS-CoV-2) has an affinity binding to angiotensin-converting-enzyme (ACE) 2 receptor, which exists in different tissues including, but not limited to, arterial and venous endothelial cells (19). As per Virchow's triad, a possible higher risk of developing thromboembolic events is expected due to vascular injury. Another possible explanation of VTE is that the inflammatory reaction caused by viral, bacterial and fungal infection results in the activation of host defense systems. This eventually contributes to the up-regulation of coagulation factors and thrombin formation pathways (20, 21). In addition to the risk factors of VTE that critically ill patients carry as a result of venous stasis (22–25), other factors that increase the risk of hypercoagulability may exist. The pro-coagulant profile, particularly D-dimer, is speculated to be a predictor of VTE development with sensitivity and specificity of 85 and 88.5%, respectively (12, 26, 27).

The anticipated benefit of increasing the anticoagulant dose is still debated. A meta-analysis of 23 retrospective observational studies found a favorable mortality reduction with the escalated dose of prophylactic anticoagulant when compared to the standard dose (28). However, high quality evidence of meta-analyses and randomized controlled trials (RCTs) investigating the clinical outcomes of various prophylactic regimens in critically ill patients have revealed conflicting results (29–33). Generally, previous studies were notably limited by poor study designs (6, 34), diversity of study populations (34–37), small sample sizes (6, 36, 38), variations in treatment settings (ICU vs. non-ICU) (39), heterogeneity of thromboprophylaxis dosing regimen definition (40), and unadjusted pooled crude estimates (34). Furthermore, numerous studies compared the effectiveness and safety of just two thromboprophylaxis regimens. However, one open question about comparing three different regimens of thromboprophylaxis simultaneously in critically ill COVID-19 patients is whether they are comparable in terms of effectiveness and coagulopathy. Obviously, limiting comparisons to two thromboprophylaxis regimens (standard and intermediate/high) may cast doubt on whether observed associations of thrombotic events or bleeding are causal or simply artifacts of more complex interrelationships between the disease itself, outcome, interventional dose, and covariates. Thus, our study aimed to assess how three different thromboprophylaxis dose regimens affect the rate of thrombotic events in critically ill COVID-19 patients.

## Methods

### Study design and setting

This retrospective analysis of the cohort study was conducted at 4 centers in Saudi Arabia, with patients hospitalized in ICUs of tertiary specialty referral hospitals: King Fahad Medical City (KFMC) in Riyadh, King Saud Medical City Hospital (KSMC) in Riyadh, Prince Mohammed Bin Abdulaziz hospital (PMAH) in Riyadh, and Almoosa Specialized Hospital in Al-Ahsa. Study approval was granted by the Institutional Review Boards at KFMC and PMAH (IRB: 20-666), KSMC (IRB:H1RI-16-Nov20-01), Almoosa hospital (IRB: ARC-20-12-4). Due to the retrospective design, informed consent was waived, as it was considered exempt. Our report adopted the STROBE (Strengthening the Reporting of Observational Studies in Epidemiology) Statement checklist (41).

### Participant selection

Lists of critically ill COVID-19 patients admitted to ICU between April 2020 and August 2021 were obtained from the health informatics officers. We used a random-selection technique to screen patients for eligibility. Random selection avoids sampling bias in giving each patient's record an equal chance of selection and coding (42). Inclusion criteria were age ≥18 years, diagnosis of critical SARS-CoV-2 infection by real-time polymerase chain reaction (RT-PCR) from the nasopharyngeal swab and receiving prophylactic anticoagulant within 24–48 h of ICU admission. Patients were excluded if pregnant, diagnosed with VTE or atrial fibrillation during COVID-19 admission, patients with chronic anticoagulants at admission, had a contraindication to anticoagulants including active bleeding, platelet counts <25 × 10^9^/L, and fibrinogen <0.5 g/L, or if they were on VTE-induced medications (oral contraceptives, tamoxifen, etc.,).

### Intensities of anticoagulant dose

This study looked at three different anticoagulant prophylactic dosing strategies: standard, intermediate, and high. The “standard dose” was defined as enoxaparin 40 mg subcutaneous (SC) daily, or 30 mg in renal failure patients, heparin 5,000 units SC twice or thrice daily, or fondaparinux 2.5 mg SC daily. The “intermediate dose” included patients treated with enoxaparin 1 mg/kg SC daily or enoxaparin 40 mg SC twice daily, or heparin 7,500 units SC twice or thrice daily. The high dose was enoxaparin 1 mg/kg SC twice daily or heparin infusion. Patients received the prescribed dose of prophylactic regimens within 24–48 h of ICU admission until hospital discharge, developing of thrombotic events, or death.

### Data collection

The study data were collected and managed using REDCap (Research Electronic Data Capture) a secure, web-based data capture application (43). Electronic case report forms (eCRFs) were developed, pilot tested, and revised accordingly. Data were manually extracted from electronic health records (EHRs) and entered into the REDCap system in a de-identified manner. A trained team of data managers were recruited to be responsible for delivering a complete, clean, and accurate dataset. Clinical data managers performed various levels of data validation following data collection, known as edit checks, until it was considered “clean” enough to support analysis. During this step, they used categories to define the essential checks which included missing data, simple range checks, logical inconsistencies, cross-form checks, and protocol violations. Edit check specification (data validation procedure) was used to ensure that all data was the same edited consistently throughout the study. Extracted data include demographic characteristics [e.g., age, gender, weight, height, body mass index (BMI)], clinical characteristics [e.g., history of VTE, diabetes, hypertension, cancer, and cerebrovascular and cardiovascular disease, renal failure, renal dialysis, post-surgery, mechanical ventilation, acute respiratory distress syndrome (ARDS), APACHE score, use of a sedative agent or paralytic agents, recent use of oral contraceptive, steroid intake, vascular access device (VAD), regimen for COVID-19 treatment], lab parameters [D-dimer, prothrombin time (PT) and activated partial thromboplastin time (APPT), international normalized ratio (INR), fibrinogen, and platelet count], clinical outcome [composite thrombotic events (PE, DVT, ischemic stroke, myocardial infarction, systemic arterial embolism)] and any bleeding (minor or major), and death.

### Study outcomes

The primary outcome was the occurrence of any component of composite thrombotic events in COVID-19 patients admitted to ICU who received standard, intermediate, or high anticoagulant doses for VTE prevention. Mortality rate and occurrence of major and minor bleeding were secondary outcomes of interest.

### Definitions

We defined severe and critical cases of COVID-19 based on the World Health Organization (WHO) (44). Severe manifestation was defined as fever plus symptoms ≥1 of the following: respiratory rate ≥30/min, dyspnea, respiratory distress, SpO_2_ ≤ 93% on room air, PaO_2_/FiO_2_ ratio <300 or lung infiltrate >50% of lung field within 24–48 h. Critical illness was evidenced by symptoms ≥1 of the following: ARDS, septic shock, altered consciousness, and/or multi-organ failure.

We defined thrombotic events as a composite outcome where at least one of the following occurred: symptomatic acute PE, DVT, ischemic stroke, myocardial infarction, or systemic arterial embolism (45). Three distinct justifications for adopting composite endpoints as the primary outcome: the rate of individual response was expected to be low; the full effect could not be captured meaningfully by a single outcome; and thrombotic events were expected to present in various manifestations of the same disease. Typically, PE diagnosis was determined by computed tomography pulmonary angiography (CTPA) or a combination of high pretest clinical probability of PE with high probability ventilation-perfusion (V/Q) lung scan. DVT diagnosis was confirmed by compression ultrasonography (CUS) with doppler of lower extremities. Myocardial infarction was diagnosed by clinical means, ECG changes and cardiac criteria according to the European Society of Cardiology (ESC) (46). Ischemic stroke was confirmed if suspected patients had brain computed tomography (CT) scan and/or magnetic resonance imaging (MRI), as well as the radiology consultant's report indicated the final diagnosis in the patient file. Mortality was defined as death that occurred during hospitalization for any reason.

Major bleeding was identified by the International Society on Thrombosis and Hemostasis (ISTH) as fatal bleeding and/or symptomatic bleeding in critical areas or organs (such as intracranial, intraspinal, intraocular, retroperitoneal, intraarticular or pericardial, or intramuscular bleeding with compartment syndrome) or documented bleeding causing a decrease in hemoglobin (Hgb) level of 2 g/dL or more, and transfusion of 2 or more units of packed red blood cells (PRBCs). In contrast, minor bleeding was all bleeds that were not considered major or non-major bleeding events (47).

### Sample size calculation

We calculated the sample size using Power Analysis and Sample Size (PASS) 11^®^ and G^*^Power (Version 3.1.9.7) software. Because of a wide variety of thrombotic events at the time of study inception, we assumed a −15% risk difference between the standard dose, intermediate dose, and high dose arms. The total sample size was calculated to be 549 with an estimated ratio of 1:1:1 (N = 183 in each arm) as needed to provide an 80% power and significance level (α, type 1 error rate) of <0.05 to assess the hypothesis.

### Statistical analysis

The three treatment groups' demographic and baseline data were compared using standard descriptive statistics. Where appropriate, continuous data were presented using means with standard deviations (±SDs) and medians with interquartile ranges (IQRs). ANOVA and Kruskal Wallis tests were used to compare normally and non-normally distributed quantitative variables between treated groups, respectively. To compare categorical variables, the chi-squared test was performed, and the results were reported as frequencies and percentages.

#### Missing data handling

Multiple Imputation by Chained Equations (MICE) approach with Nelson Aalen estimator was used to handle missing data, which was regarded as missing at random (MAR) (48, 49). To accommodate for uncertainty, multiple dataset predictions (5 imputed datasets with 10 iterations) were constructed for each missing value in this method, resulting in decreased variability and more accurate standard errors. Only variables with <15% missing data were considered. Convergence and density plots were visually checked for missing variables. All estimates were pooled across the generated datasets.

#### Matching weights procedure and survival analysis

Due to the challenge of multiple covariates and multiple treatment arms in this observational study, we applied the matching weights method, an extension of inverse probability of treatment weighting (IPTW), as a sensitivity analysis to estimate the average treatment effect (ATE) outcome across multiple treatment groups (50). The approach reweights all standard, intermediate, and high groups to simulate a propensity score-matched population. Multinomial logistic regression was used to fit all propensity scores of the covariates of interest. The use of this methodology is explained by Yoshida et al. (50). Post-weighting balance assessment is used to find the optimal balance with absolute standardized mean differences of <0.2 for all covariates, indicating better covariate balance (50). Propensity score distribution and overlap was visually inspected *via* a mirror diagram. A covariate balance check was demonstrated *via* a Love plot. To estimate the probability of survival of the three groups, we used an adjusted Kaplan-Meier (KM) model with weighted data. Time 0 was the time of anticoagulation initiation. Censoring was considered for patients with no events that were discharged alive or were still admitted patients at the time of data collection. The stratified log-rank test was used to compare the survival distributions of three samples. In order to draw weighted KM curves, we used “svykm” function from the “survey” package in R which assumes weights as sampling weights to account for the matching design. This would allow the estimation of robust standard errors for survival. For the relative effect, we fitted the Cox regression model. The assumptions were adequately checked by using Schoenfeld residuals and visual plots. We implemented robust variance estimation to estimate standard errors. In the case of low to no events, we could not estimate hazards ratios.

#### Statistical software

R Core Team (51) software (R Foundation for Statistical Computing, Version 4.0.1, Vienna, Austria) was used. The following packages in the R interface were used to conduct the analyses: survival (52), survey (53), mice (48), MatchThem (54), cobalt (55) and tableone (56).

## Results

### Baseline characteristics

**Figure 1:**
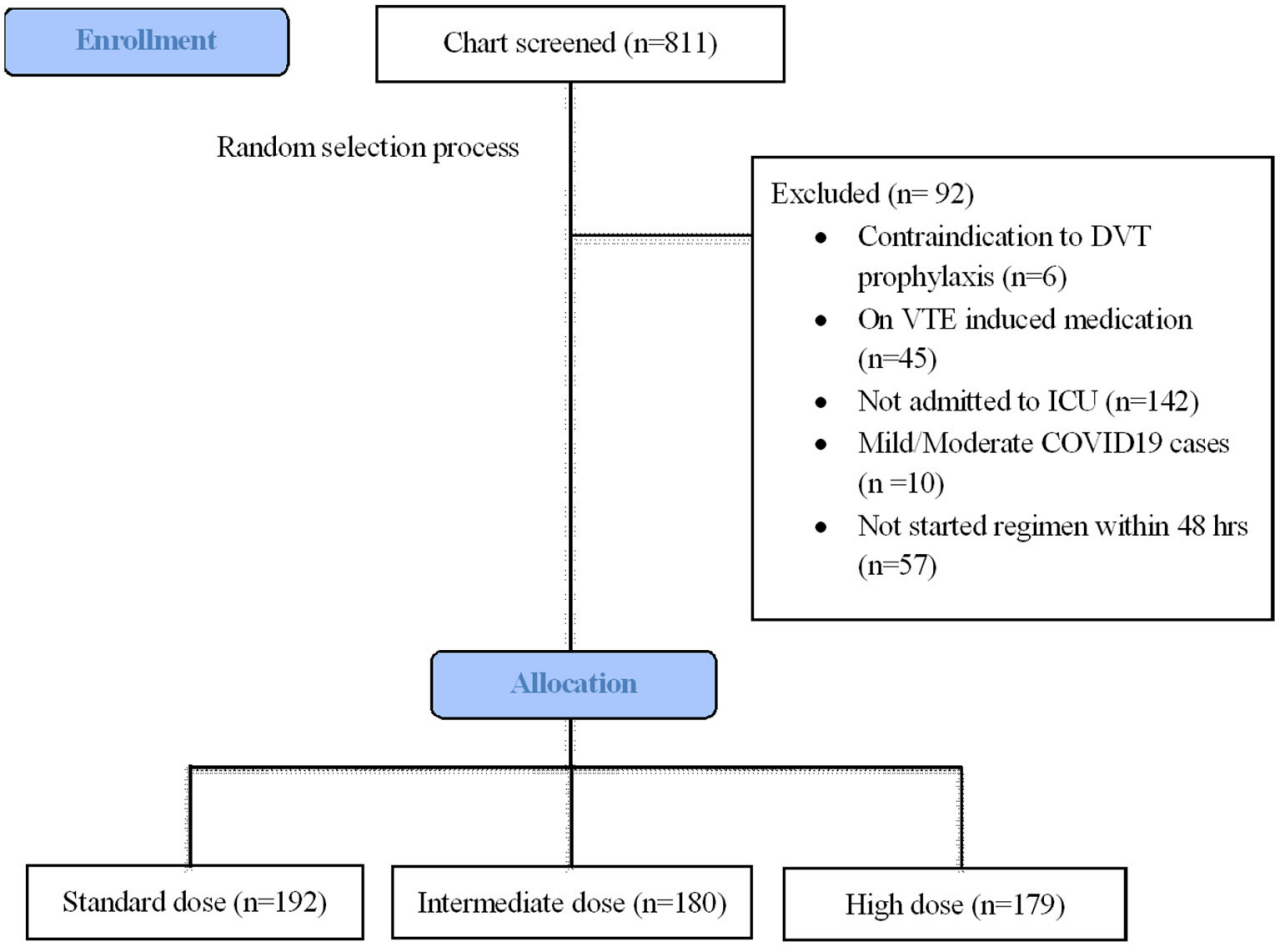
Patients selection flowchart.

During the study period, 811 patients were screened for eligibility criteria (Figure 1). A total of 551 patients met inclusion criteria for analysis, with the remaining patients being excluded due to lack of ICU admission (n = 142), late start of thromboprophylaxis regime >48 h (n = 57), anticoagulant contraindications (n = 6), mild/moderate COVID-19 cases (n = 10), and on VTE-induced medicine (n = 6). Table 1 summarizes the main baseline characteristics of the standard dose group (n = 192), intermediate-dose group (n = 180), and high dose group (n = 179). A total of 112 (58%) patients out of the 192 standard dosing patients received heparin; whereas only 16 (8.9%) received heparin intermediate dosing and only 1 patient received heparin high dosing. None of this cohort received fondaparinux. For the pre-covariate balance of original data, there were explicit differences in the baseline characteristics of age (*P* = 0.032), gender (*P* = 0.017), ethnicity (*P* = 0.006), kidney function (*P* < 0.001) APACHE II score (*P* = 0.009), WHO severity (*P* < 0.001), mechanical ventilation (*P* = 0.001), and medications such as intravenous steroid (*P* = 0.001) and carbapenem (*P* = 0.021). Additionally, groups differed in the following laboratory variables: d-dimer (*P* = 0.014) and APTT (*P* < 0.001). Missing data were highest for fibrinogen (37.9%), followed by APACHE II score and D-dimer (7–10%) (Table 1, footnote).

**Table 1:** Baseline characteristics.

**Variable**	**Standard** **(*n* = 192)**	**Intermediate** **(*n* = 180)**	**High** **(*n* = 179)**	***P* value**
Age, mean (SD)	59.2 (15.0)	56.4 (13.8)	55.6 (13.1)	0.032
Female, n (%)	61 (31.8)	39 (21.7)	36 (20.1)	0.017
Ethnicity				0.006
Middle eastern	113 (58.9)	75 (41.7)	89 (49.7)	
Southeast/east Asian	8 (4.2)	14 (7.8)	12 (6.7)	
South Asian	26 (13.5)	46 (25.6)	39 (21.8)	
Central Asian	8 (4.4%)	9 (5.0)	0 (0.0)	
African	15 (7.8)	7 (3.9)	12 (6.7)	
Other/Unknown	23 (12.0)	28 (15.6)	27 (15.1)	
Weight (kg), median (IQR)	77.5 (67.5–90.0	78.6 (70.0–90.0)	80.0 (70.0–90.0)	0.141
BMI (kg/m^2^), median (IQR)	27.9 (24.8–31.8	28.0 (24.8–31.8	28.0 (24.8–32.4)	0.849
Scr (mg/dl), median (IQR)	1.4 (0.8–2.6)	1.0 (0.7– 2.1)	1.0 (0.8–1.4)	0.003
CKD EPI (mL/min/m^2^), median (IQR)	53.0 (22.5–93.6)	79.3 (32.9–101.2)	79.4 (47.9–99.4)	<0.001
CKD stage, n (%)				<0.001
Stage 1	54 (28.1)	73 (40.6)	64 (35.8)	
Stage 2	30 (15.6)	37 (20.6)	47 (26.3)	
Stage 3a	17 (8.9)	8 (4.4)	23 (12.8)	
Stage 3b	21 (10.9)	13 (7.2)	18 (10.1)	
Stage 4	43 (22.4)	27 (15.0)	15 (8.4)	
Stage 5	20 (10.4)	15 (8.3)	4 (2.2)	
Unknown	7 (3.6)	7 (3.9)	8 (4.5)	
APACHE II score, median (IQR)	18.0 (12.0–23.0)	15.0 (10.0–22.0)	14.0 (10.0–21.0)	0.009
PT (s), median (IQR)	14.2 (13.0–16.0)	14.0 (12.9–15.7)	14.6 (13.2–16.1)	0.058
INR, median (IQR)	1.2 (1.0–1.3)	1.1 (1.0–1.3)	1.1 (1.1–1.3)	0.229
APTT (s), median (IQR)	34.0 (29.9–47.0)	32.0 (28.0–38.0)	35.0 (29.4–42.4)	<0.001
Platelet (per 109/L), median (IQR)	229.0 (145.0–323.0)	249.0 (184.0–340.0)	244.0 (184.0–340.0)	0.074
Fibrinogen (g/L), median (IQR)	4.9 (3.3–6.7)	4.9 (3.2–6.6)	5.3 (3.2–7.4)	0.395
D-Dimer (μg/ml), median (IQR)	1.9 (1.0–4.4)	2.0 (0.9– 6.0)	2.8 (1.3–7.4)	0.014
Respiratory disease, n (%)	28 (14.6)	21 (11.7)	20 (11.2)	0.559
Established Cardiovascular disease, n (%)	20 (10.4)	17 (9.4)	22 (12.3)	0.674
Diabetes, n (%)	110 (57.3)	96 (53.3)	95 (53.1)	0.655
Hypertension, n (%)	108 (56.2)	82 (45.6)	90 (50.3)	0.117
Dyslipidemia, n (%)	11 (5.7)	10 (5.6)	15 (8.4)	0.476
Liver disease, n (%)	5 (2.6)	1 (0.6)	3 (1.7)	0.313
HIV status, n (%)	3 (1.6)	1 (0.6)	0 (0.0)	0.331
Mechanical ventilation, n (%)	144 (75.0)	117 (65.0)	90 (50.3)	<0.001
ECMO, n (%)	28 (14.6)	26 (14.4)	10 (5.6)	<0.001
WHO severity				
Severe	47 (24.5)	70 (38.9)	91 (50.8)	<0.001
Critical	145 (75.5)	110 (61.1)	88 (49.2)	<0.001
Sedative agent, n (%)	173 (90.1)	165 (91.7)	162 (90.5)	0.911
COVID-19 regimen during hospital stay, n (%)				
Favipiravir	20 (10.4)	28 (15.6)	31 (17.3)	0.132
Remdesivir	3 (1.6)	6 (3.3)	3 (1.7)	0.140
Hydroxychloroquine	4 (2.1)	5 (2.8)	4 (2.2)	0.939
Azithromycin	3 (1.6)	6 (3.3)	12 (6.7)	0.037
Lopinavir/ritonavir	7 (3.6)	8 (4.4)	3 (1.7)	0.341
Hydroxychloroquine+azithromycin	0 (0.0)	2 (1.1)	2 (1.1)	0.398
Azithromycin + Favipiravir	2 (1.0)	2 (1.1)	5 (2.8)	0.482
Azithromycin + Favipiravir + Tocilizumab	2 (1.0)	5 (2.8)	9 (5.0)	0.075
Hydroxychloroquine+Azithromycin+Tocilizumab	0 (0.0)	5 (2.8)	0 (0.0)	0.007
Lopinavir/ritonavir+Interferon-B+Ribavirin	5 (2.6)	9 (5.0)	9 (5.0)	0.043
IV steroid use, n (%)	162 (84.4)	168 (93.3)	175 (97.8)	<0.001
ACEI or ARB, n (%)	33 (17.2)	22 (12.2)	33 (18.4)	0.234
Beta blocker, n (%)	64 (33.3)	51 (28.3)	72 (40.2)	0.057
Calcium Channel Blocker, n (%)	65 (33.9)	55 (30.6)	59 (33.0)	0.783
Aspirin, n (%)	52 (27.1)	46 (25.6)	52 (29.1)	0.757
Insulin, n (%)	141 (73.4)	132 (73.3)	122 (68.2)	0.443
Statin, n (%)	68 (35.4)	54 (30.0)	48 (26.8)	0.191
Thiazide diuretic, n (%)	2 (1.0)	2 (1.1)	4 (2.2)	0.609
Loop diuretic, n (%)	82 (42.7)	94 (52.2)	107 (59.8)	0.004
Cefepime, n (%)	19 (9.9)	12 (6.7)	23 (12.8)	0.143
Vancomycin, n (%)	112 (58.3)	92 (51.1)	100 (55.9)	0.366
Carbapenems, n (%)	103 (53.6)	105 (58.3)	121 (67.6)	0.021
Piperacillin/Tazobactam, n (%)	107 (55.7)	103 (57.2)	82 (45.8)	0.061
Metronidazole, n (%)	6 (3.1)	4 (2.2)	2 (1.1)	0.445

*BMI, body mass index. Scr, serum creatinine. CKD-EPI, Chronic Kidney Disease Epidemiology Collaboration. The Acute Physiology and Chronic Health Evaluation (APACHE II), Established cardiovascular disease was defined as a documented history of stable angina, unstable angina, percutaneous coronary intervention (PCI), coronary artery bypass graft surgery, or myocardial infarction (MI), heart failure or cerebrovascular disease included transient ischemic attack (TIA) or stroke. Respiratory disease, asthma or chronic obstructive pulmonary disease (COPD). HIV, human immunodeficiency virus. ECMO, extracorporeal membrane oxygenation. ACEI, angiotensin-converting enzyme inhibitors. WHO, World health organization. ARB, angiotensin receptor blockers. CCB, calcium channel blockers. IV, intravenous. Carbapenems included meropenam and imipenam.*

*Missing data: <1% (paralytic agent, INR, vasopressor use, BMI, steroid use, weight in kg). 1–5% (treatment regimen during hospitalization, PT, APTT, platelet count, Scr, CKD EPI). 7–10% (APACHE II score, D-dimer). Fibrinogen (37.9%)*.

**Figure 2:**
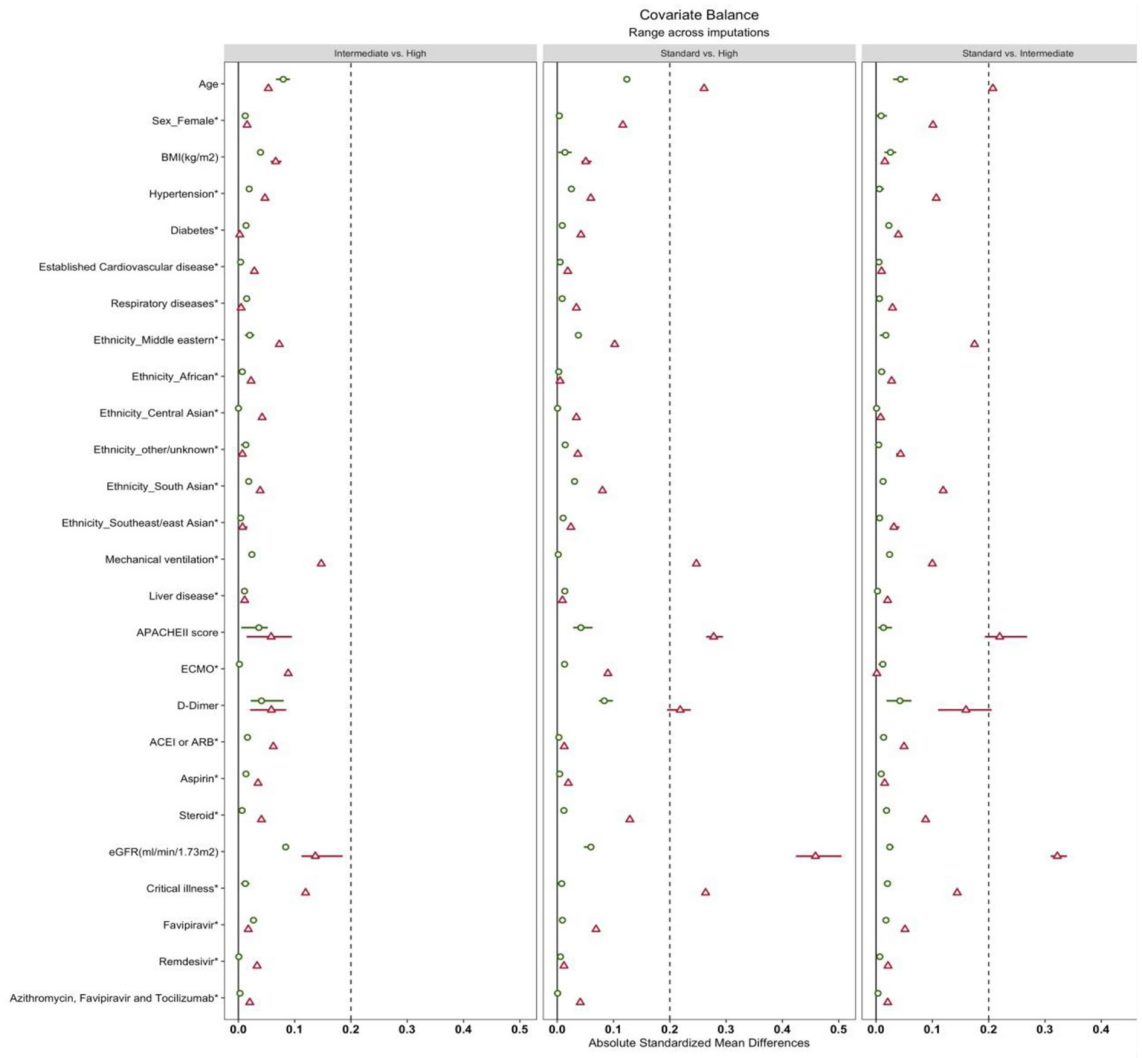
Love plots of covariate balance. BMI, body mass index. Established cardiovascular disease was defined as a documented history of stable angina, unstable angina, percutaneous coronary intervention (PCI), coronary artery bypass graft surgery, myocardial infarction (MI), heart failure or cerebrovascular disease included transient ischemic attack (TIA) or stroke. Respiratory disease: asthma or chronic obstructive pulmonary disease (COPD), the Acute Physiology and Chronic Health Evaluation (APACHE II). ECMO, extracorporeal membrane oxygenation; ACEI, angiotensin-converting enzyme inhibitors; ARB, angiotensin receptor blockers; eGFR, estimated Glomerular Filtration Rate.

After applying the weights matching procedure, all covariates of interest were adequately balanced (Table 2; Figure 2). We illustrated the propensity score distribution using a Love plot for absolute standardized mean difference (SMD) distribution (Figure 2). Most patients (>60%) had D-dimer >1.5 μg/ml (Supplementary Table S1).

**Table 2:** Covariate balance post weight matching procedure and multiple imputations.

**Covariate**	**Pre-covariate balance**			**Post-covariate balance**				
**Data**	**Original data**			**Weight matching**				
**Arm**	**Standard**	**Intermediate**	**High**	**Absolute**	**Standard**	**Intermediate**	**High**	**Absolute SMD**
	***n* = 192**	***n* = 180**	***n* = 179**	**SMD**	***n* = 102**	***n* = 103**	***n* = 104**	
**Age, mean (**±**SD)**	59.2 (14.98)	56.4 (13.79)	55.6 (13.12)	0.169	56.43 (14.99)	57.07 (13.66)	58.06 (12.75)	0.079
**Female**, ***n*** **(%)**	61 (31.8)	39 (21.7)	36 (20.1)	0.179	23.7 (23.4)	26.0 (25.3)	24.4 (23.4)	0.030
**Ethnicity**, ***n*** **(%)**				0.401	29.18 (6.54)	29.35 (6.06)	29.11 (6.31)	0.070
Middle Eastern	113 (58.9)	75 (41.7)	89 (49.7)		56.2 (55.3)	56.0 (54.6)	53.9 (51.8)	
Central Asian	7 (3.6)	9 (5.0)	0 (0.0)		0.0 (0.0)	0.0 (0.0)	0.0 (0.0)	
East/Southeast Asian	8 (4.2)	14 (7.8)	12 (6.7)		6.3 (6.2)	5.7 (5.6)	5.4 (5.2)	
South Asian	26 (13.5)	46 (25.6)	39 (21.8)		17.8 (17.6)	18.9 (18.4)	21.2 (20.4)	
African	15 (7.8)	8 (4.4)	12 (6.7)		6.6 (6.5)	7.0 (6.9)	7.0 (6.7)	
Unknown/other	23 (12.0)	28 (15.6)	27 (15.1)		14.7 (14.4)	15.0 (14.6)	16.6 (16.0)	
**BMI (kg/m** ^**2**^ **), mean (SD)**	28.95 (6.37)	28.85 (6.02)	29.25 (6.08)	0.043	29.18 (6.54)	29.35 (6.06)	29.11 (6.31)	0.025
**CKD EPI (ml/min/m** ^**2**^ **) mean (SD)**	61.54 (40.50)	73.53 (39.79)	77.84 (43.87)	0.263	70.25 (40.70)	71.17 (40.48)	67.70 (34.65)	0.061
**Respiratory diseases**, ***n*** **(%)**	28 (14.6)	21 (11.7)	20 (11.2)	0.068	13.2 (12.9)	14.0 (13.6)	12.6 (12.1)	0.030
**Established cardiovascular diseases**, ***n*** **(%)**	20 (10.4)	17 (9.4)	22 (12.3)	0.061	12.2 (12.0)	11.2 (10.9)	11.3 (10.9)	0.032
**Type 1 or 2 diabetes**, ***n*** **(%)**	110 (57.3)	96 (53.3)	95 (53.1)	0.057	50.7 (49.7)	55.5 (50)	57.5 (55.3)	0.036
**Hypertension**, ***n*** **(%)**	108 (56.2)	82 (45.6)	90 (50.3)	0.143	50.7 (49.7)	51.5 (50)	54.5 (52.4)	0.032
**Liver disease**, ***n*** **(%)**	5 (2.6)	1 (0.6)	3 (1.7)	0.112	0.7 (0.7)	1.0 (1.0)	2.1 (2.0)	0.078
**Mechanical ventilation**, ***n*** **(%)**	144 (75.0)	117 (65.0)	90 (50.3)	0.350	70.5 (69.0)	68.5 (66.8)	71.9 (61.1)	0.036
**ECMO**, ***n*** **(%)**	28 (14.6)	26 (14.4)	10 (5.6)	0.201	11.0 (10.8)	9.6 (9.3)	9.9 (9.5)	0.034
**WHO critical category**, ***n*** **(%)**	145 (75.5)	110 (61.1)	88 (49.2)	0.374	64.8 (36.5)	67.7 (66.0)	67.4 (64.7)	0.032
**D-dimer (μg /mL), mean (SD)**	5.11 (10.37)	7.00 (14.62)	7.60 (12.74)	0.136	5.20 (11.30)	5.49 (10.60)	6.00 (8.44)	0.053
**APACHE II score, mean (SD)**	18.45 (8.42)	16.70 (8.46)	15.70 (8.56)	0.217	16.70 (8.48)	16.57 (8.64)	16.95 (8.99)	0.029
**ACEI or ARB**, ***n*** **(%)**	33 (17.2)	22 (12.2)	33 (18.4)	0.115	17.3 (17.0)	16.1 (15.7)	18.0 (17.3)	0.029
**Aspirin**, ***n*** **(%)**	52 (27.1)	46 (25.6)	52 (29.1)	0.052	28.7 (28.1)	28.4 (27.6)	29.7 (28.6)	0.013
**Favipiravir**, ***n*** **(%)**	20 (10.4)	28 (15.6)	31 (17.3)	0.134	14.3 (14.1)	12.8 (12.4)	15.4 (14.8)	0.046
**Remdesivir**, ***n*** **(%)**	3 (1.6)	7 (3.9)	1 (0.6)	0.156	1.5 (1.5)	0.8 (0.8)	1.0 (1.0)	0.044
**Steroid**, ***n*** **(%)**	164 (85.4)	170 (94.4)	176 (98.3)	0.333	97.3 (95.4)	100.5 (97.9)	101.1 (79.2)	0.084
**Azithromycin, favipiravir and tocilizumab**, ***n*** **(%)**	2 (1.0)	5 (2.8)	9 (5.0)	0.159	2.0 (2.0)	1.6 (1.6)	2.0 (1.9)	0.021

*BMI, body mass index. Scr, serum creatinine. CKD-EPI, Chronic Kidney Disease Epidemiology Collaboration. Established cardiovascular disease was defined as a documented history of stable angina, unstable angina, percutaneous coronary intervention (PCI), coronary artery bypass graft surgery, or myocardial infarction (MI). Heart failure and cerebrovascular disease included transient ischemic attack (TIA) or stroke. Respiratory disease: asthma or chronic obstructive pulmonary disease (COPD). VTE, venous thromboembolism. ECMO, extracorporeal membrane oxygenation; ARB, angiotensin receptor blockers; WBC, White blood cells; Hgb, Hemoglobin; Pao_2_, partial pressure of oxygen; FiO2, fraction of inspired oxygen; ALT, Alanine transaminase; ALP, Alkaline phosphatase; CRP, c-reactive protein; IQR, Interquartile range; SMD: Standardized mean difference. Percentages were rounded.*

*Prior Weight matching, missing data were imputed using Multivariate Imputation by Chained Equations (MICE) equations that included Nelson–Aalen estimator*.

### Thrombotic events and mortality outcomes

**Figure 3:**
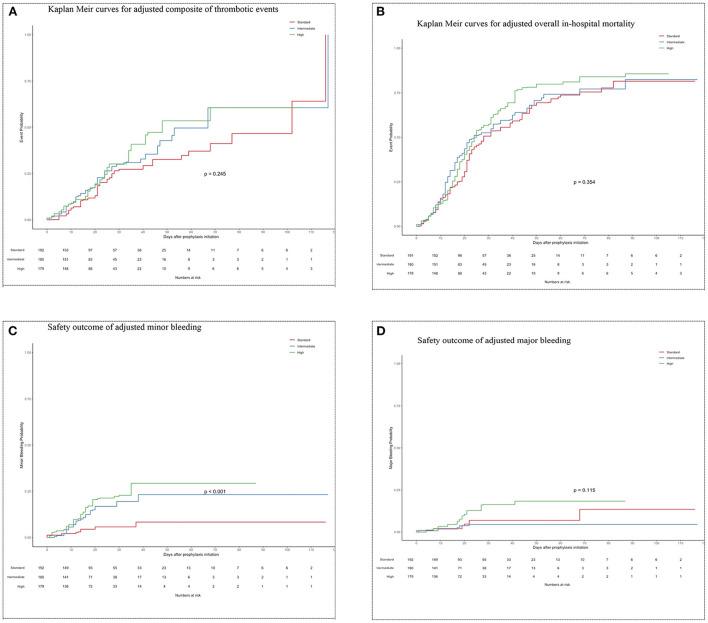
**(A)** Kaplan Meir curves for adjusted composite of thrombotic events. **(B)** Kaplan Meir curves for adjusted overall in-hospital mortality. **(C)** Safety outcome of adjusted minor bleeding. **(D)** Safety outcome of adjusted major bleeding.

After weights matching, the risk of a composite of thrombotic events was not significantly different between the standard-dose and intermediate-dose groups {21.6 vs. 25.2%; adjusted hazard ratio (aHR) = 1.4, [95% confidence of interval (CI), 0.88–2.33]} (Table 3; Figure 3A). Also, the standard-dose group was associated with a similar hazard of thrombotic events to the high-dose group [21.6 vs. 28.8%; aHR = 1.3 (95% CI, 0.83–2.20)] (Table 3; Figure 3A). Furthermore, each component of the composite outcome has comparative events in the three different prophylactic anticoagulant intensities (Table 3). A subgroup of patients having a D-dimer of >1.5 vs. <1.5 g/ml were associated with similar composite thrombotic events (Supplementary Table S1). For secondary outcome, patients on standard-dose and intermediate-dose arms had comparable overall in-hospital mortality [51.0 vs. 53.4%; aHR = 1.2 (95% CI, 0.88 to 1.72)] (Table 3; Figure 3B). Similarly, we found in-hospital mortality occurred more frequently in the high-dose groups (61.1%) than in the standard-dose group (51.0%), although the findings were not statistically significant [aHR = 1.3 (95% CI, 0.92–1.74)] (Table 3; Figure 3B). When the unadjusted hazard ratio was considered, analyses revealed no differences in primary outcome and mortality (Supplementary Figures S1A,B).

**Table 3:** Clinical outcomes results.

**Outcome, n (%)**	**Standard**	**Intermediate**	**HR**	**High**	**HR (95%CI)^†^**
	**(n = 192)**	**(n = 180)**	**(95%CI)^†^**	**(n = 179)**	
**(A) Unadjusted outcomes**					
**Composite endpoint**	38 (19.8)	45 (25.0)	1.46 (0.94–2.26)	43 (24.0)	1.31(0.85–2.04)
Pulmonary embolism	25 (13.0)	28 (15.6)	1.37 (0.79–2.35)	30 (16.8)	1.42 (0.83–2.43)
Deep venous thrombosis	13 (6.8)	5 (2.8)	0.49 (0.17–1.40)	5 (2.8)	0.40 (0.14–1.15)
Stroke	0 (0.0)	5 (2.8)	0.91(0.30–2.77)	4 (2.2)	0.92 (0.30–2.78)
Myocardial infarction	|3 (1.6)	7 (3.9)	2.87 (0.74–11.15)	9 (5.0)	3.63 (0.98–13.47)
Systemic arterial embolism	0 (0.0)	0 (0.0)	–	1 (0.6)	–
**Hospital death**	112 (58.3)	93 (51.7)	1.18 (0.85–1.66)	104 (58.1)	
**Bleeding**					
Minor	10 (5.2)	22 (12.2)	2.66 (1.26–5.64)	31 (17.3)	3.73 (1.82–7.63)
Major	6 (3.1)	6 (3.3)	0.23 (0.41–3.93)	18 (10.1)	3.81 (1.51–9.65)
**Outcome, n (%)**	**Standard**	**Intermediate**	**HR**	**High**	**HR (95%CI)** ^†^
	**(*****n*** = **102)**	**(*****n*** = **103)**	**(95%CI)** ^†^	**(*****n*** = **104)**	
**(B) Matching weights procedure**					
**Composite endpoint**	22 (21.6)	26 (25.2)	1.4 (0.88–2.33)	30 (28.8)	1.3 (0.83–2.20)
Pulmonary embolism	13 (12.7)	16 (15.3)	1.3 (0.71–2.46)	22 (21.2)	1.7 (0.90–3.02)
Deep venous thrombosis	8 (7.8)	3 (2.9)	0.4 (0.12–1.29)	3 (2.9)	0.3 (0.10–1.12)
Stroke	0 (0.0)	3 (2.9)	1.2 (0.33–4.05)	3 (2.9)	0.8 (0.23–2.98)
Myocardial infarction	2 (2.0)	5 (4.9)	3.2 (0.68–0.53)	5 (4.8)	2.8 (0.61–12.59)
Systemic arterial embolism	0 (0.0)	0.0 (0.0)	–	1.0 (1.0)	–
**Hospital death**	52 (51.0)	55 (53.4)	1.2 (0.88–1.72)	64 (61.1)	1.3 (0.92–1.74)
**Bleeding**					
Minor	5 (4.9)	13 (12.6)	2.9 (1.26–6.80)	18 (17.3)	3.9 (1.73–8.76)
Major	5 (4.9)	3 (2.9)	0.8 (0.23–2.74)	9 (8.6)	2.1 (0.79–5.80)

*Occurrence of any composite endpoint defined as symptomatic acute pulmonary embolism (PE), deep-vein thrombosis, ischemic stroke or myocardial infraction. Follow up day is to the first event occurred.*

*^†^All comparisons were against standard as reference.*

*^a^Odds ratios were estimated.*

*Bleeding score according to International Society on Thrombosis and Haemostasis bleeding scale.*

*HR, Hazard ratio; CI, Confidence intervals*.

### Bleeding outcome

Minor bleeding in the intermediate-dose group (12.6%) was higher compared to the standard-dose group (4.9%) [aHR = 2.9 (95% CI, 1.26–6.80)] (Table 3; Figure 3C). Also, higher proportion of patients experienced minor bleeding in high-dose group (17.3%) vs. the standard-dose group (4.9%) [aHR = 3.9 (95% CI, 1.73–8.76)] (Table 3; Figure 3C). No significant differences regarding major bleeding were observed between the 3 study groups (standard: 4.9 vs. intermediate: 2.9 vs. high: 8.6%) (Table 3; Figure 3D). The unadjusted analysis can be seen in Table 3 and Supplementary Figures S1C,D).

## Discussion

This retrospective cohort study compared three different thromboprophylactic regimens in COVID-19 ICU patients. We found that these three regimens (standard-dose, intermediate-dose, and high-dose groups) had comparable primary composite of thrombotic events, including the major components. Furthermore, no significant differences were observed between the three study groups with respect to hospital mortality. Those who received intermediate-dose and high-dose groups had a similar frequency of major bleeding events as those who received standard-dose. However, there were more minor bleeding events in the intermediate-dose and high-dose groups compared with the standard-dose group.

The effectiveness of anticoagulant dose escalation in COVID-19 ICU patients continues to be debated, even among RCT studies. Our findings are in line with the hypothesis that high-dose thromboprophylaxis has a similar composite of thrombotic events in critically ill patients with COVID-19 as intermediate-dose and standard-dose thromboprophylaxis regimens (29, 31, 32). In addition, parallel to observational and RCT studies (29, 31, 32, 57, 58), different thromboprophylaxis doses did not eliminate the risk of overall mortality. This cast doubt on other studies that show a superior survival rate with escalated doses (37, 59, 60). Of note, a meta-analysis of observational studies, has also confirmed that increasing the anticoagulation regimen to the therapeutic dose, resulted in an increase in bleeding events (28).

To the best of our knowledge, only one study investigated the efficacy of three different anticoagulation dose regimens in COVID-19-infected critically ill patients (60). It concluded that high-dose thromboprophylaxis was associated with a lower risk of cumulative incidence of thromboembolic events and fewer bleeding events compared with lower doses. However, the study's findings should be interpreted with caution because of study limitations that may lead to misleading estimates of treatment effect. This includes small sample size and that almost half (45.4%) of patients underwent dose adjustment of the anticoagulant during ICU stay (60).

There are several possible explanations for the high incidence of venous thromboembolism (VTE) in the ICU population. One possible explanation is that the bioavailability of subcutaneous thromboprophylaxis is reduced, especially in edematous patients or those who receive vasoactive medications concurrently, thereby potentially providing reduced efficacy (61). Another possibility is low cardiac output in a population with pre-existing cardiovascular disease (62). Furthermore, significant VTE occurrence was observed not only with COVID-19, but also with other epidemic respiratory virus infections. When relevant data from previous virus infections is reviewed, it provides a lesson from the past about the magnitude of coagulation disorders' severity when compared to COVID-19 infection. Critically ill patients with H1N1 virus and SARS-CoV-1 showed substantial VTE rates of 44 and 30%, respectively (63, 64).

Some studies speculated that abnormal coagulation parameters, such as elevated D-dimer in COVID-19 (D-dimer > 1.5 μg/ml), were predictors of ICU admission, mortality and the development of VTE (12, 13, 65–67). However, the generalizability of these results is subject to certain limitations. First, those studies were limited by a small sample size, lack of serial D-dimer monitoring, absence of laboratory methodology details for the D-dimer assay, and lack of validation. Also, in one study, continuous D-dimer data was empirically categorized (levels of ≤ 0.5 μg/ml, >0.5 to ≤ 1 μg/ml, and >1 μg/ml) instead of using receiver operating characteristic (ROC) analysis to determine the optimal cutoff predictive value of D-dimer for poor prognosis and mortality (67). Second, the D-dimer assay test is limited by low specificity and high rate of false-positive results in a variety of non-thrombotic conditions, such as inflammation, infection, sepsis, female gender, black race, increased aging, active malignancy, sickle cell disease, lupus, chronic liver disease, trauma or surgical status (68). Third, compared to the current and previous pandemic and epidemic viruses, we noticed that D-dimer levels were elevated in both severely infected COVID-19 and SARS-CoV-1 patients, at 59.6 and 45%, respectively (69, 70). Thus, using D-dimer levels as a marker to inform anticoagulant dosing regimens in ICU patients may be inadequate for clinical decision-making.

The definition of prophylactic anticoagulation intensity played a vital role in determining the extent to which study results would be affected (71). Notably, dosing regimens of anticoagulant varied widely across different studies, where it was defined according to local site protocols or trial protocol (28, 33). The therapeutic dose in the large REMAP-CAP/ACTIV-4a/ATTACC multiplatform trial was defined as to meet the target for aPTT of 1.5 to 2.5 times the upper limit of normal (for unfractionated heparin) or therapeutic anti-Xa levels (for enoxaparin). (32). The study concluded that therapeutic dose was associated with significantly lower rates of VTE and higher rates of major and minor bleeding (32). However, these results were inconsistent with what we found. Considering that thrombotic events were not regularly screened during hospital stay in our institutions, but rather, were prompted by the treating physician upon suspicion. This would underestimate the rate of thrombotic events in our cohort. However, given local protocol-based practice, this would be in line with current COVID-19 treatment guidelines panel recommendations (71).

In addition, we noted in our study significant variations in heparin and enoxaparin proportions between groups. Comparing how each anticoagulant type might affect the magnitude of clinical outcomes was beyond the scope of this study. Additionally, practical constraints might prevent designing different study groups with various anticoagulant dosing regimens and types. Obviously, a still open question is whether the anticoagulant type will affect the efficacy and safety outcomes.

Many studies compare just two different thromboprophylaxis regimens. Our study shares many features with others, but the combination used is unique. First, it represents a comprehensive examination of the most frequent three different thromboprophylaxis dosing regimens used in ICU admitted COVID-19 patients. Being familiar with the same efficacy of three prophylactic-dose regimes empowers clinicians to make decisions and recommend a standard dose of thromboprophylaxis in non-obese ICU COVID-19 patients. Second, this study was conducted at 4 centers (multicenter) in two Saudi Arabian cities. Third, in our analysis, we considered all possible covariates that may influence the thrombotic events and mortality findings for ICU patients when developing the Cox regression prediction model. Fourth, this study offers valuable insight into high-risk patients, such as those with elevated D-dimer levels, with patients receiving standard, intermediate, and high-dose thromboprophylaxis, having mean D-dimer baselines of 5.65, 6.44, and 7.54 μg/ml, respectively.

The American Society of Hematology guidelines and Saudi Critical Care Society practice guidelines suggest using the standard prophylactic dose for adults with critical COVID-19 who had no clinical suspicion of VTE (72, 73). However, our findings may counter the uncertainty about using standard prophylactic dose in patients with high suspicion of VTE, namely those with D-dimer level >1.5 μg/ml (in >60% of our cohort). More research using randomized controlled trials is needed to investigate the efficacy and safety of three different anticoagulation regimens in critically ill COVID-19 patients with D-dimer levels >1.5 μg/ml.

The present study was subject to a several potential weaknesses. First, it was an observational cohort study, which could have included unmeasured confounding factors that could not be accounted for without a randomized study design. Second, despite many of our patients having D-dimer levels above 1.5 μg/ml, around 30–35% of each group had D-dimer levels <1.5 μg/ml, which gives further caution on the generalizability of these findings in this populations of patients. Lastly, missing data could bias our estimates, despite using the powerful statistical tool MICE.

## Conclusion

This current study shows that standard, intermediate, high anticoagulation dose targets for thromboprophylaxis of critically ill COVID-19 patients have a comparable composite of thrombotic events and mortality. An escalated dose of thromboprophylaxis (intermediate and high dose) could increase the rate of minor bleeding but not major bleeding when compared to the standard dose. Thus, these data recommend the standard-dose as preferred regimen.

## Data availability statement

The original contributions presented in the study are included in the article/Supplementary material, further inquiries can be directed to the corresponding author/s.

## Ethics statement

The study approval was granted by the Institutional Review Boards at KFMC and PMAH (IRB: 20-666), KSMC (IRB: H1RI-16-Nov20-01), and Almoosa Hospital (IRB: ARC-20-12-4). Written informed consent for participation was not required for this study in accordance with the national legislation and the institutional requirements.

## Author contributions

Conceptualization was done by AAlr, YM, and AAlam. Statistics, methodology, software, and interpretation of the results were done by AAlam and AAlr. Study supervision was done by PC. Consultation on statistics was provided by PC and IA. The first draft of the paper was done and the abstract was drafted by AAlr. All authors contributed to the final version of the manuscript.

## Conflict of interest

The authors declare that the research was conducted in the absence of any commercial or financial relationships that could be construed as a potential conflict of interest.

## Publisher's note

All claims expressed in this article are solely those of the authors and do not necessarily represent those of their affiliated organizations, or those of the publisher, the editors and the reviewers. Any product that may be evaluated in this article, or claim that may be made by its manufacturer, is not guaranteed or endorsed by the publisher.
