# Supplementary material for: A comparison of three thromboprophylaxis regimens in critically ill COVID-19 patients: An analysis of real-world data
Source: Front Cardiovasc Med. 2022 Aug 16;9:978420. doi: 10.3389/fcvm.2022.978420 (PMC9424612; doi:10.3389/fcvm.2022.978420)
Supplement: Supplementary file 1 [file Data_Sheet_1.pdf]

Supplements Content

- Figure S1. A) Kaplan Meir curves for unadjusted composite of thrombotic events. B) Kaplan Meir curves for unadjusted overall in-hospital mortality. C) Safety outcome of unadjusted minor bleeding. D) Safety outcome of unadjusted major bleeding
- Table S1. Dose interaction with D-dimer

Figure S1A: Kaplan Meir curves for unadjusted composite of thrombotic events

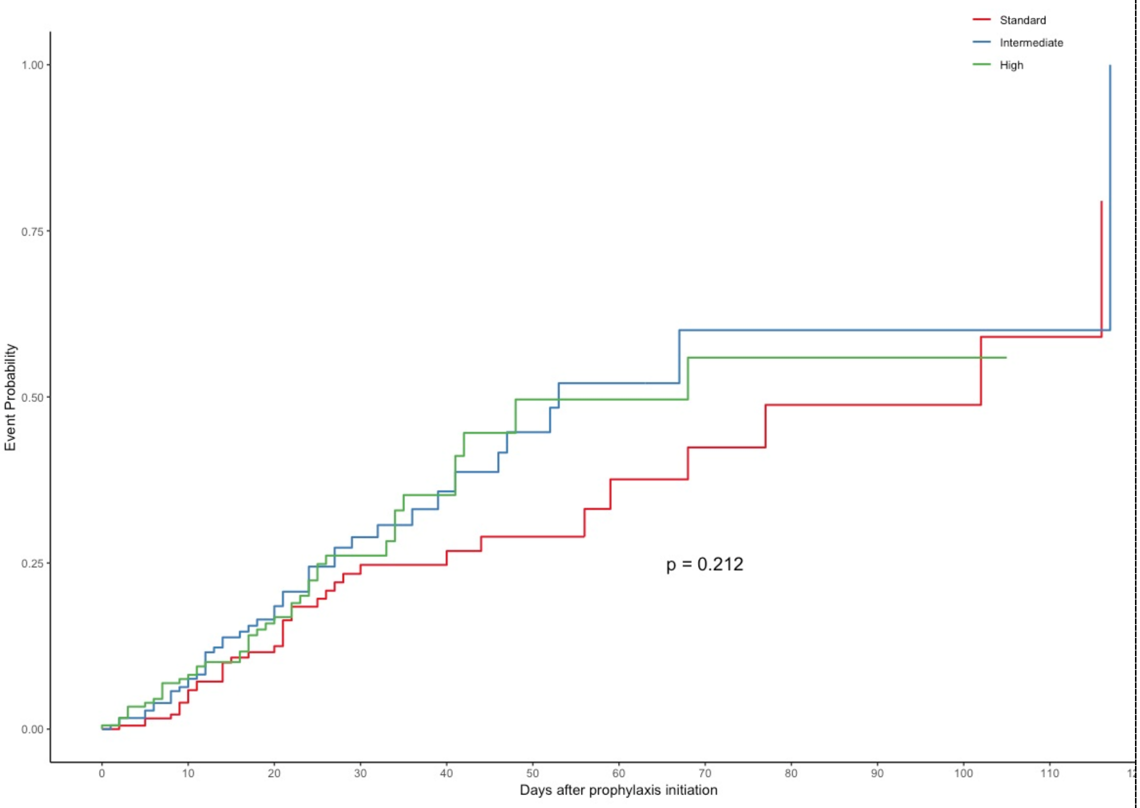

Standard	192	153	97	57	36	25	14	11	7	6	6	2
ntermediate	180	151	83	45	23	16	8	3	3	2	1	1
High	179	148	88	43	22	10	9	6	6	5	4	3

Numbers at risk

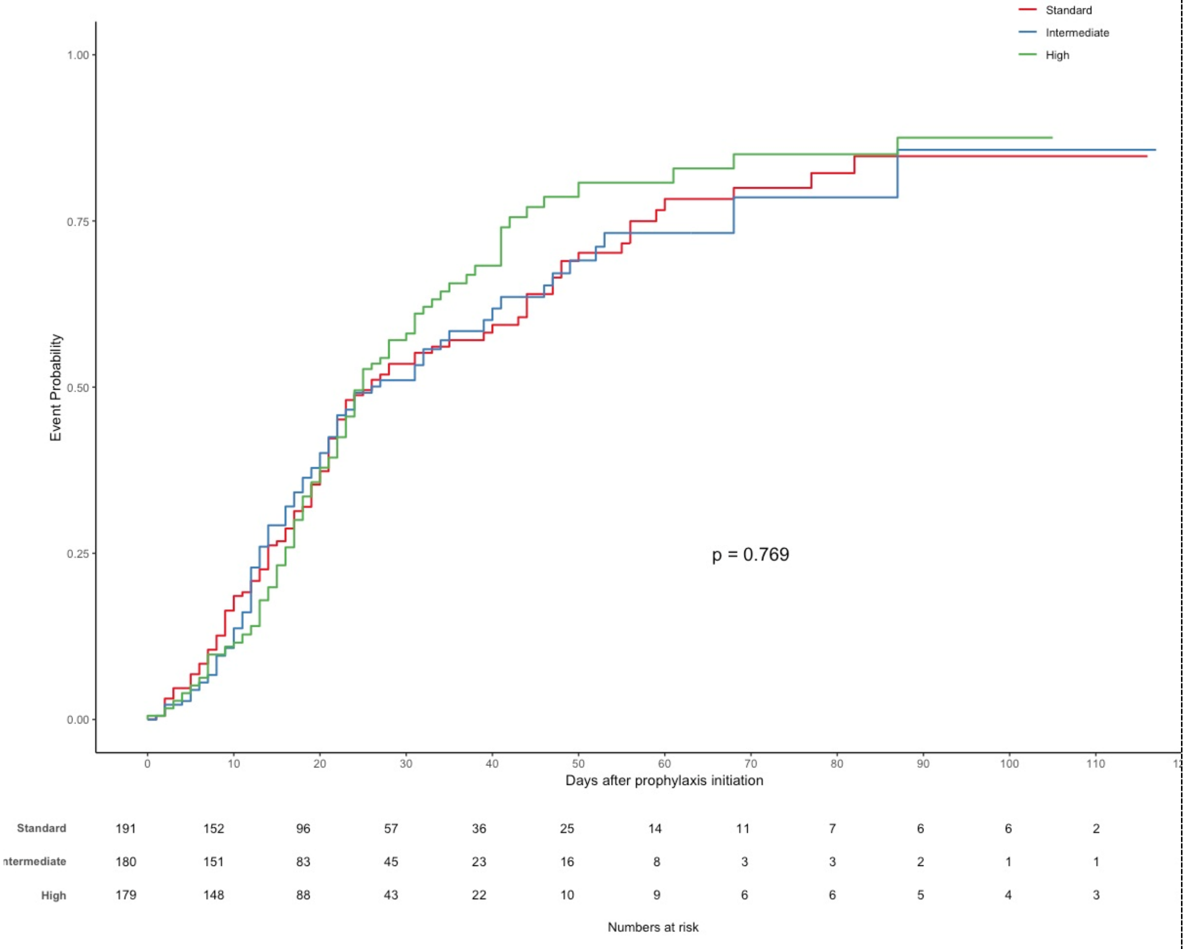

Figure S1B: Kaplan Meir curves for unadjusted overall in-hospital mortality

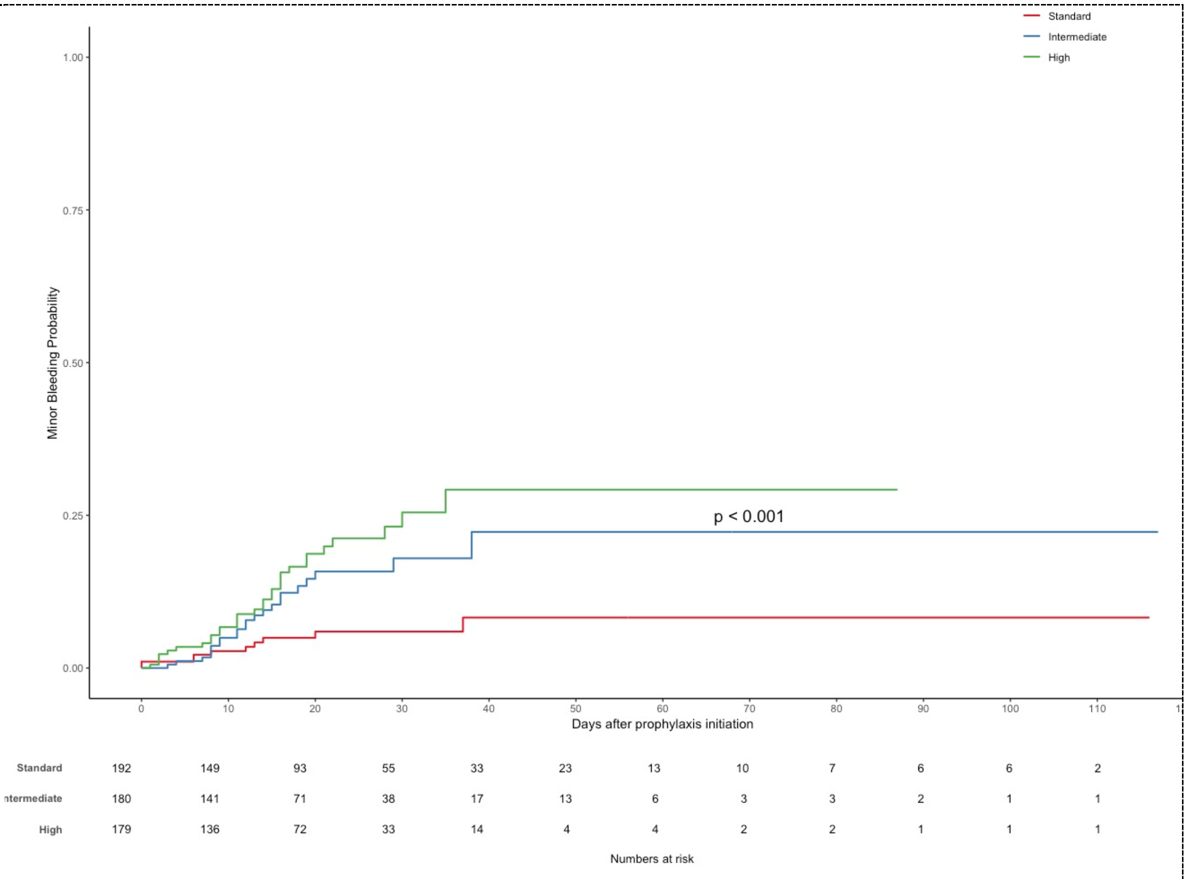

Figure S1C: Safety outcome of unadjusted minor bleeding

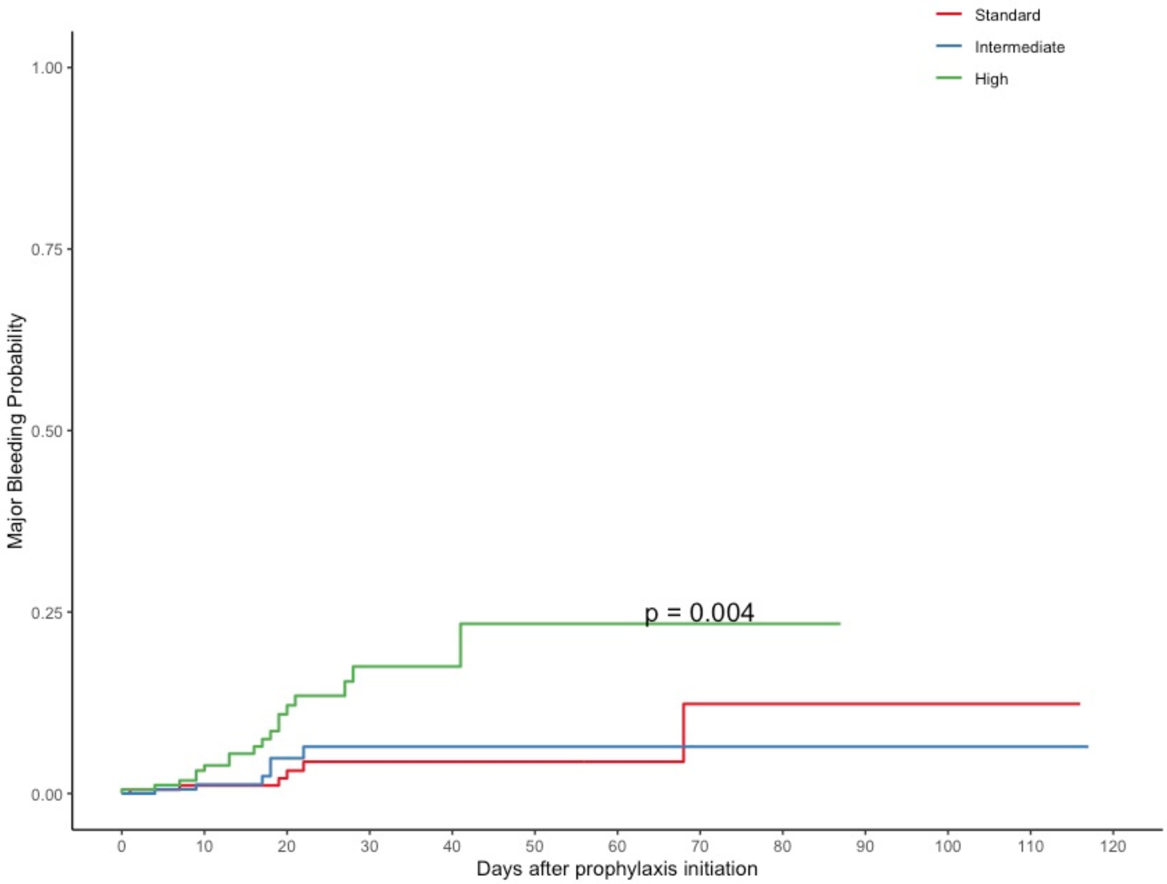

Standard	192	149	93	55	33	23	13	10	7	6	6	2	1
ntermediate	180	141	71	38	17	13	6	3	3	2	1	1	0
High	179	136	72	33	14	4	4	2	2	1	1	1	1

Numbers at risk

Figure S1D: Safety outcome of unadjusted major bleeding

Table S1. Dose interaction with D-dimer			
Variable	Standard dose (n=102)	Intermediate dose (n=103)	High Dose (n=104)
D-dimer **(µg /mL)**			
>= 1.5, n (%)	63 (62.4)	65 (63.4)	73 (70.0)
Patients with composite primary outcome (n, %)	12.1 (19.0)	15.6 (23.9)	22.0 (30.2)
Odds ratios (95% CI)	Reference	1.19 (0.39 to 3.69)	1.93 (0.57 to 6.53)
CI: Confidence interval			
